# Copy Number Variation in the Horse Genome

**DOI:** 10.1371/journal.pgen.1004712

**Published:** 2014-10-23

**Authors:** Sharmila Ghosh, Zhipeng Qu, Pranab J. Das, Erica Fang, Rytis Juras, E. Gus Cothran, Sue McDonell, Daniel G. Kenney, Teri L. Lear, David L. Adelson, Bhanu P. Chowdhary, Terje Raudsepp

**Affiliations:** 1 Department of Veterinary Integrative Biosciences, College of Veterinary Medicine, Texas A&M University, College Station, Texas, United States of America; 2 School of Molecular and Biomedical Science, The University of Adelaide, Adelaide, South Australia, Australia; 3 New Bolton Center, School of Veterinary Medicine, University of Pennsylvania, Pennsylvania, United States of America; 4 Ontario Veterinary College, University of Guelph, Guelph, Ontario, Canada; 5 M.H. Gluck Equine Research Center, Veterinary Science Department, University of Kentucky, Lexington, Kentucky, United States of America; 6 New Research Complex, Qatar University, Al Tarfa, Doha, Qatar; Stanford University School of Medicine, United States of America

## Abstract

We constructed a 400K WG tiling oligoarray for the horse and applied it for the discovery of copy number variations (CNVs) in 38 normal horses of 16 diverse breeds, and the Przewalski horse. Probes on the array represented 18,763 autosomal and X-linked genes, and intergenic, sub-telomeric and chrY sequences. We identified 258 CNV regions (CNVRs) across all autosomes, chrX and chrUn, but not in chrY. CNVs comprised 1.3% of the horse genome with chr12 being most enriched. American Miniature horses had the highest and American Quarter Horses the lowest number of CNVs in relation to Thoroughbred reference. The Przewalski horse was similar to native ponies and draft breeds. The majority of CNVRs involved genes, while 20% were located in intergenic regions. Similar to previous studies in horses and other mammals, molecular functions of CNV-associated genes were predominantly in sensory perception, immunity and reproduction. The findings were integrated with previous studies to generate a composite genome-wide dataset of 1476 CNVRs. Of these, 301 CNVRs were shared between studies, while 1174 were novel and require further validation. Integrated data revealed that to date, 41 out of over 400 breeds of the domestic horse have been analyzed for CNVs, of which 11 new breeds were added in this study. Finally, the composite CNV dataset was applied in a pilot study for the discovery of CNVs in 6 horses with XY disorders of sexual development. A homozygous deletion involving *AKR1C* gene cluster in chr29 in two affected horses was considered possibly causative because of the known role of *AKR1C* genes in testicular androgen synthesis and sexual development. While the findings improve and integrate the knowledge of CNVs in horses, they also show that for effective discovery of variants of biomedical importance, more breeds and individuals need to be analyzed using comparable methodological approaches.

## Author Summary

Genomes of individuals in a species vary in many ways, one of which is DNA copy number variation (CNV). This includes deletions, duplications, and complex rearrangements typically larger than 50 base-pairs. CNVs are part of normal genetic variation contributing to phenotypic diversity but can also be pathogenic and associated with diseases and disorders. In order to distinguish between the two, detailed knowledge about CNVs in the species of interest is needed. Here we studied the genomes of 38 normal horses of 16 diverse breeds, and identified 258 CNV regions. We integrated our findings with previously published horse CNVs and generated a composite dataset of ∼1400 CNVRs. Despite this large number, our analysis shows that CNV research in horses needs further improvement because the current data are based on 10% of horse breeds and that most CNVRs are study-specific and require validation. Finally, we analyzed CNVs in horses with disorders of sexual development and found in two male pseudo-hermaphrodites a large deletion disrupting a group of genes involved in sex hormone metabolism and sexual differentiation. The findings underline the possible role of CNVs in complex disorders such as development and reproduction.

## Introduction

The significance of gene duplication in long-term evolutionary changes was already recognized over 40 years ago by Susumu Ohno [1]. Yet, systematic genome-wide discovery and functional interpretation of inter- and intraspecific copy number variations (CNVs) in genes and non-genic DNA sequences, started in the past decade with foundational studies in humans [2], [3] and mice [4], followed by genome-wide (GW) CNV discovery in chicken [5], cattle [6], dogs [7], [8] and other domestic species (see [9], [10]). It is now well established that CNVs are a common feature of vertebrate genomes. Typically, they are DNA sequence variants from at least 50 base-pairs (bp) to over several megabase-pairs (Mb) in size that are involved in deletions, insertions, duplications and translocations, causing structural differences between genomes [11], [12]. In terms of the total number of DNA base-pairs, CNVs are responsible for more heritable sequence differences (0.5–1%) between individuals than SNPs (0.1%) [11], [12], [13].

One of the central goals of CNV research has been determining their association with genome instability, genetic diseases and congenital disorders. It is thought that CNVs, as a major source of inter-individual genetic variation, could explain variable penetrance of Mendelian and polygenic diseases, and variation in the phenotypic expression of complex traits [14], [15]. Indeed, CNVs have been associated with common complex and polygenic disorders in humans affecting a broad range of biological processes, such as immune response, autoimmunity and inflammation [3], [16], [17]; musculoskeletal [18], [19] and cardiovascular systems [20], [21]; neurodevelopment, cognition and behavior [22], [23], and sexual development and reproduction [24], [25], [26], [27], [28].

The availability of whole genome (WG) sequence draft assemblies combined with the advances in array-based technologies and next generation sequencing (NGS), have prompted CNV research in all main domestic animal species (reviewed by [9], [10]) with the most advanced information currently available for cattle [6], [29], [30], pigs [31], and dogs [32], [33], [34].

In horses, five studies report about the discovery of CNVs in the whole genome [35], [36], [37], [38] or in gene exons [39]. Attempts have also been made to associate CNVs with equine diseases [36], adaptations [38] and phenotypic traits [37], [39]. While these studies set a foundation for understanding the role of CNVs in equine biology, the current information is inadequate for efficient discovery of variants affecting equine health and disorders. This is because the studies have used different CNV discovery platforms, the number of breeds and individuals in some studies is very limited, and the majority of reported CNVs are study-specific and not validated by two or more independent studies. Also, the available information has not been integrated into a composite dataset to facilitate the analysis of known CNVs and the discovery of new ones.

The aim of this study is to improve the current rather limited knowledge of CNVs in horses by their genome-wide discovery in multiple individuals of additional diverse horse breeds. Using a custom-made WG tiling array we generate a CNV map for the horse genome and integrate this with the previous CNV studies into a composite dataset. Finally, we carry out a pilot CNV analysis in horses with disorders of sexual development to test the utility of the array and the integrated dataset for the discovery of variants involved in equine complex disorders.

## Results

### The Texas-Adelaide horse whole-genome tiling array

Texas A&M University (USA) and The University of Adelaide (Australia) collaborated to create a whole-genome (WG) 400K tiling array which was produced and printed by Agilent Technologies (Design ID #030025), and designated as the Texas-Adelaide array. The probes on the array represented 18,763 autosomal and X-linked genes, and intergenic, sub-telomeric and chrY sequences. Median genomic distance between the probes on the array was 7.5 kb; this distance was lower (4 kb) in sub-telomeric regions, and higher (∼20 kb) in the Y chromosome. Before using the array for CNV discovery in horses, the platform was tested for performance quality. Self-to-self control hybridizations (Figure S1a) showed 1.55% of False Discovery Rate (FDR) - an indication that the array design, fabrication, and array genomic hybridization (aCGH) procedures were optimal. As a proof-of principle, female-to-male hybridizations between two half-sib Thoroughbreds, *Twilight* (female) and *Bravo* (male), showed massive loss in the X chromosome and a gain in the Y chromosome in the male, whereas only one CNV was detected in an autosome, chr3 (Figure S1b). Hybridization quality was assessed by measuring Derivative Log Ratio Standard Deviation (DLRSD) which calculates probe-to probe log ratio noise and is typically <0.3 for good quality hybridizations. The DLRSD values for all hybridizations with blood DNA from *Twilight* and *Bravo* were <0.2. Therefore, and because the oligonucleotides on the array were derived from the sequences of these two horses, DNA of *Twilight* and *Bravo* was used as a reference for all aCGH experiments: *Twilight* for females and *Bravo* for males. Further, because our DNA collection from horse breeds contained samples isolated from blood and hair, an additional self-to-self hybridization was conducted using DNA from blood and hair of one male Quarter Horse QH3-H528 (Table S1). Blood DNA gave good quality results with DLRSD  = 0.14, whereas consistent and high level hybridization noise was observed for hair DNA (DLRSD  = 0.41) (Figure S1c). Due to this, CNVs in all samples were called with stringent criteria: log_2_ ratio alterations higher than 0.5 over 5 neighboring probes – a necessary compromise between calling CNVs with confidence and missing a few true calls. With median probe spacing of 7.5 kb on the array, this allowed detection CNVs of about 30 kb, and in probe-dense regions even smaller. We concluded that the performance of the equine 400K Texas-Adelaide whole-genome CGH array was optimal for the discovery of CNVs in the horse genome.

### CNV discovery and construction of a whole-genome CNV map for the horse

The aCGH data are available at NCBI GEO accession GSE55266. Collectively, 950 CNV calls were made across 36 horses, with an average of 26.4 calls per individual (Table 1; Table S3). The number of CNV calls was the highest in two American Miniature Horses (59 and 46) and the lowest in American Quarter Horses (12 and 14), whereas the number of calls per individual was not significantly different between blood and hair DNA (*P* = 0.07; Table 1) at the settings of log_2_±0.5 over 5 probes. The number and distribution of CNVRs in the two Przewalski horses were similar to those in domestic horses (Table 1, Table S4). Because the Thoroughbred served as a reference, by default all the 950 CNV calls recorded in other breeds were also present in the Thoroughbred, though inversely with respect to gains and losses. However, because the Thoroughbred was compared with multiple individuals, the same CNV had different log_2_ values, and that is why the Thoroughbreds were not included in the comparisons of CNV metrics.

**Table 1:** Breed- and individual-wise summary of CNV calls in horses.

Horse breed	Source of DNA for aCGH	CNV calls per individual	Gains	Losses
Akhal-teke 1	Blood	37	12	25
Akhal-teke 2	Blood	26	13	13
American Miniature Horse 1	Blood	59	16	43
American Miniature Horse 2	Hair	46	4	42
American Quarter Horse 1	Blood	12	0	12
American Quarter Horse 2	Blood	21	2	19
American Quarter Horse 3	Blood	14	10	4
Arabian 1	Blood	21	17	4
Arabian 2	Hair	17	0	17
Belgian 1	Blood	31	14	17
Belgian 2	Hair	14	1	13
Caspian Pony 1	Blood	40	16	24
Caspian Pony 2	Hair	12	1	11
Clydesdale 1	Blood	25	6	19
Clydesdale 2	Hair	16	1	15
Exmoor Pony 1	Blood	29	15	14
Exmoor Pony 2	Hair	18	8	10
Fell Pony 1	Blood	25	11	14
Fell Pony 2	Hair	47	11	36
Friesian 1	Blood	29	6	23
Friesian 2	Blood	39	10	29
Friesian 3	Blood	41	9	32
Friesian 4	Blood	22	12	10
Mongolian Native Horse1	Hair	22	1	21
Mongolian Native Horse2	Hair	18	2	16
Percheron 1	Blood	17	11	6
Percheron 2	Hair	12	1	11
Przewalski's Horse 1	Fibroblasts	21	5	16
Przewalski's Horse 2	Fibroblasts	21	3	18
Sorraia 1	Blood	36	8	28
Sorraia 2	Hair	18	1	17
Standardbred 1	Blood	17	7	10
Standardbred 2	Blood	44	13	31
Swiss Warmblood 1	Blood	23	1	22
Swiss Warmblood 2	Blood	30	6	24
Swiss Warmblood 3	Blood	29	9	20
Thoroughbred 1	Blood	Male reference	n/a	n/a
Thoroughbred 2	Blood	Female reference	n/a	n/a
**Average**		**26.4**	**7.3**	**19.1**
**Median**		**22.5**	**7.5**	**17.0**

The number of calls per individual was not significantly different (Student's T-test *p* = 0.07) between hair and blood DNA.

The ADM-2 algorithm arranged adjacent and overlapping CNV calls (CNVs) within and between individual horses into 258 CNV regions (CNVRs; Table S5) of which 114 were shared between at least 2 individuals of the same or different breeds, while 144 were private and found only in one individual. Two CNVRs were found in two or more individuals of the same breed but not in other breeds and were tentatively considered as breed-specific: a 14 kb loss in chr9 in Exmoor ponies, and a 39 kb loss in chr20 in Swiss Warmblood horses (Table S4).

**Figure 1:**
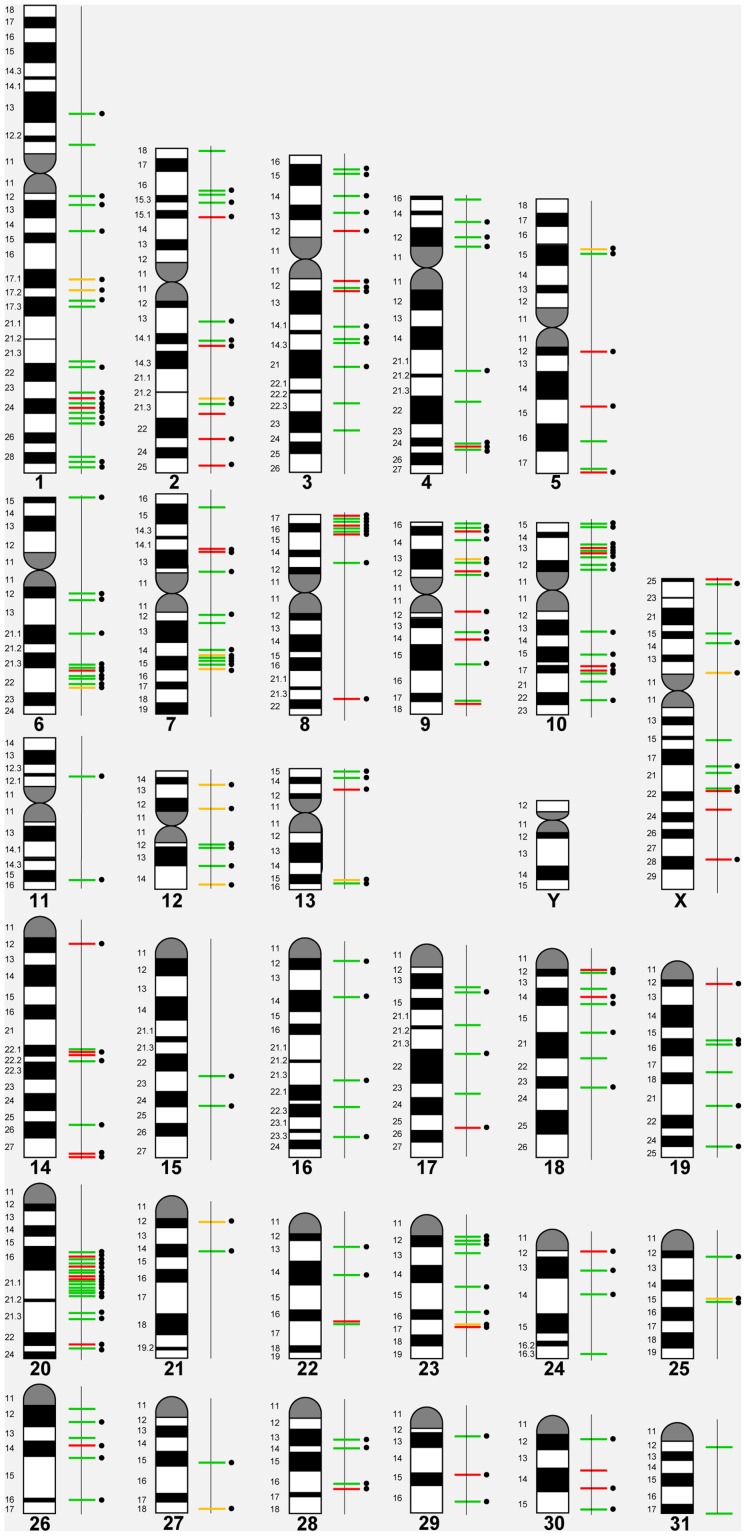
A CNVR map of the horse genome. Green line – loss; red line – gain; yellow line – complex; black dots – genes involved.

Based on the 258 CNVRs, a whole genome CNV map for the horse was constructed (Figure 1) details of which are summarized in Table 2. The mean size of CNVRs was 110 kb ranging from 1 kb to 2.5 Mb. The CNVRs occupied 1.15 % of the equine genome and were distributed over all horse chromosomes, except the Y, with the highest enrichment in chromosomes 12 (9.7%) and 20 (3.0 %). Even though chr12 is the gene richest chromosome in the horse genome (15 genes/Mb), there was no overall correlation between CNV enrichment and gene density. For example, the enrichment values for the second and third gene densest chromosomes, chr11 and chr13, were 0.02% and 0.28%, respectively (Table 2). Likewise, we did not observe CNV enrichment in sub-telomeres, as previously reported for humans [40]: the array contained 5,716 sub-telomeric probes, though only 10 CNVRs were detected in these regions in horses.

**Table 2:** Chromosome-wise CNVR statistics for the horse genome.

Chr	#CNVR	Shared	Private	Novel	Gains	Losses	Complex	Genic	Intergenic	Sub-telomeric	Mean CNVR size (bp)	CNVR length (bp)	Chr. size (bp)	Enrich-ment, %	Gene/Mb
1	21	8	13	9	2	17	2	14	7	0	241,828	5,078,379	185,838,109	2.73	6.63
2	13	3	10	5	5	7	1	9	4	1	69,711	906,246	120,857,687	0.75	6.64
3	14	3	11	3	3	11	0	11	3	0	80,328	1,124,591	119,479,920	0.94	5.19
4	9	4	5	3	1	8	0	7	2	0	119,780	1,078,019	108,569,075	0.99	4.94
5	7	5	2	2	3	3	1	4	3	2	60,379	422,653	99,680,356	0.42	8.14
6	11	5	6	3	1	9	1	9	2	0	206,650	2,273,151	84,719,076	2.68	9.08
7	12	4	8	3	2	8	2	8	4	0	119,155	1,429,861	98,542,428	1.45	9.36
8	9	5	4	1	4	5	0	8	1	1	170,025	1,530,227	94,057,673	1.63	5.66
9	14	6	8	11	5	8	1	4	10	1	45,785	640,986	83,561,422	0.77	5.66
10	16	7	9	7	4	12	0	13	3	1	72,954	1,167,268	83,980,604	1.39	8.16
11	2	0	2	1	0	2	0	2	0	0	5,235	10,470	61,308,211	0.02	14.71
12	6	5	1	1	0	3	3	5	1	0	538,645	3,231,871	33,091,231	9.77	14.97
13	5	2	3	1	1	3	1	5	0	0	23,837	119,184	42,578,167	0.28	11.69
14	8	3	5	3	5	3	0	5	3	1	55,523	444,184	93,904,894	0.47	5.41
15	2	0	2	2	0	2	0	2	0	0	80,429	160,857	91,571,448	0.18	5.32
16	5	3	2	4	0	5	0	4	1	0	46,120	230,599	87,365,405	0.26	5.95
17	6	2	4	3	1	5	0	3	3	0	100,527	603,159	80,757,907	0.75	3.1
18	8	4	4	4	2	6	0	5	3	0	59,831	478,649	82,527,541	0.58	3.66
19	6	1	5	5	1	5	0	2	4	0	69,818	418,908	59,975,221	0.70	5.23
20	19	11	8	4	5	14	0	13	6	0	102,575	1,948,920	64,166,202	3.04	8.85
21	2	1	1	1	0	1	1	2	0	1	230,842	461,684	57,723,302	0.80	4.87
22	4	2	2	1	1	3	0	2	2	0	48,296	193,182	49,946,797	0.39	8.08
23	8	2	6	4	1	6	1	5	3	0	122,812	982,492	55,726,280	1.76	3.91
24	4	1	3	2	1	3	0	3	1	0	104,966	419,862	46,749,900	0.90	6.19
25	3	2	1	1	0	2	1	2	1	0	46,573	139,720	39,536,964	0.35	10.56
26	6	4	2	3	1	5	0	2	4	0	105,913	635,479	41,866,177	1.52	3.68
27	2	1	1	0	0	1	1	2	0	0	15,084	30,168	39,960,074	0.08	3.91
28	4	2	2	2	1	3	0	2	2	0	37,557	150,227	46,177,339	0.33	6.42
29	3	2	1	2	1	2	0	3	0	0	250,105	750,316	33,672,925	2.23	4.17
30	4	1	3	2	2	2	0	2	2	0	67,956	271,825	30,062,385	0.90	4.23
31	2	1	1	2	0	2	0	0	2	1	34,286	68,572	24,984,650	0.27	4.58
X	12	5	7	10	4	7	1	0	12	1	87,146	1,045,753	124,114,077	0.84	4.46
Un	11	9	2	11	6	0	5	0	11	0	4,118	45,298	117,461,955	0.04	-
Y	0	0	0	0	0	0	0	0	0	0	0	0	0	0.00	0.57
**Total**	**258**	**114**	**144**	**116**	**63**	**173**	**22**	**158**	**100**	**10**	**110,437**	**28,492,760**	**2,484,515,402**	**1.15**	**-**

Shared – found in 2 or more individuals; private – in one horse only; novel – not reported before; the horse genome statistics was retrieved from Ensembl (http://www.ensembl.org/index.html).

In general, losses (173; 67%) prevailed over gains (63; 24%), although 6 horses had more gains than losses (Table 1). Twenty-two CNVRs (8.5%) were complex involving both losses and gains in different individuals (Table 2, Table S3). Even though aCGH on diploid samples cannot discriminate between copies of alleles and thus, distinguish between heterozygous and homozygous CNVs, two gains and 14 losses were tentatively considered homozygous because of log_2_ alterations over 2.0 (Table S6). Homozygosity of 8 losses was confirmed by qualitative PCR (Fig. S2).

### Gene content of CNVRs and functional categories of copy number variable genes

**Figure 2:**
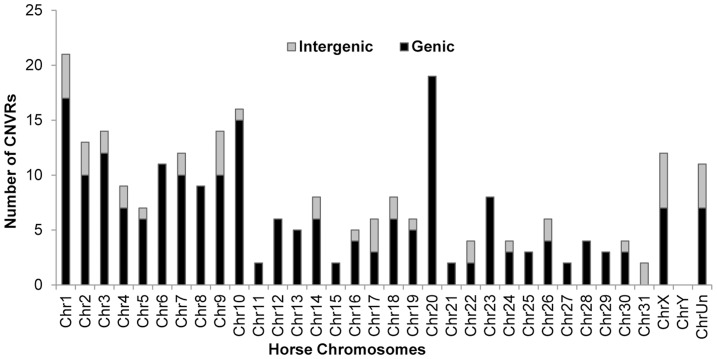
Chromosome-wise distribution of genic and intergenic CNVRs in the horse genome.

The majority (82%) of horse CNVRs contained one or more known Ensembl (http://www.ensembl.org/index.html) horse genes (158 CNVRs) or non-horse mammalian reference genes (54 CNVRs) (Table S7), while 46 CNVRs (18%) were located in intergenic regions (Table S8). Gene containing CNVRs were also predominant in individual chromosomes with the exception of chr31 which was enriched with intergenic variants Fig. 2. However, we consider calls for intergenic CNVRs tentative and subject to change as the annotation of the horse genome is still in progress.

**Figure 3:**
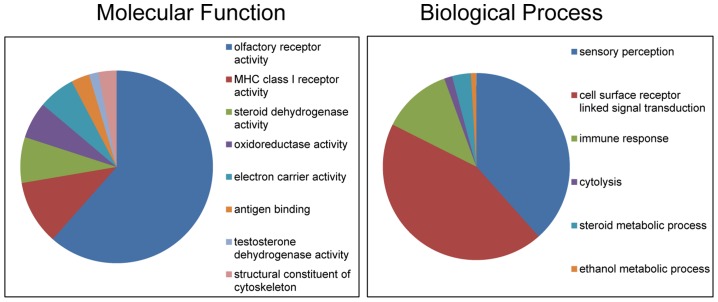
Gene Ontology classifications of copy number variable genes in horses.

Altogether, the CNVRs involved 805 protein-coding genes (750 Ensembl genes, 33 non-Ensembl genes and 22 horse mRNAs; Table S7) but also non-coding small and long RNA genes, and pseudogenes. The largest CNVRs with the highest number of genes corresponded to clusters of olfactory and non-olfactory G-protein coupled receptors (GPCRs) or to immunity related genes, such as immunoglobulins, T-cell receptors, and MHC protein complex genes - a typical feature of CNVRs in all mammalian genomes studied so far [3], [30], [32], [39], [41], [42]. Likewise, Gene Ontology (GO) analysis indicated that equine copy number variable genes are predominantly involved in biological processes and molecular functions related to transmembrane signal transduction, chemo-attractant sensory perception, immune response and steroid metabolism (Fig. 3; Table S9). Notably, 5 copy number variable genes from this study were associated with known OMIA (http://omia.angis.org.au/home/) phenotypes for immune, reproductive or neuromuscular diseases (Table 3), though none of the OMIA records involved horses or CNVs. The CNVR overlapping with the *BMPR1B* gene has been earlier reported in horses and is of interest because of a possible role in the regulation of the rate of ovulation [39].

**Table 3:** Equine copy number variable genes with known mammalian phenotypes (OMIA; http://omia.angis.org.au/home/).

Gene symbol	Biological system, phenotype and mutation	OMIA ID	Ensembl ID	Location Chr:Mb	CNVR size, kb	CNVR Type	Discovery breeds	Reference for CNVR
*BMPR1B*	*REPRODUCTIVE*: Fecundity in Booroola and Bonpala sheep; missense mutation	000383–9940; 001423–9940	ENSECAG 00000012140	3: 43.57–43.60	28	Loss	Friesian, Quarter Horse, Standardbred, Swiss Warmblood	This study, [39]
*BTN1A1*	*IMMUNE*: Resistance to avian sarcoma and leucosis viruses in chicken; nonsense mutation	001622–9031	ENSECAG 00000017948	20:24.22–24.62	405	Gain	American Miniature, Arabian, Belgian, Caspian, Clydesdale, Fell Pony, Friesian, Standardbred, Sorraia, Swiss Warmblood	This study, [36], [38], [39]
*CFH*	*IMMUNE*: Membranoproliferative glomerulonephritis type II in pigs; missense mutation	000636–9825	ENSECAG 00000011534	30:24.74–24.87	132	Loss	Sorraia, Clydesdale, Fell Pony, Friesian, Standardbred, Swiss Warmblood	This study
*GLB1*	*NEUROMUSCULAR*: Gangliosidosis in sheep, cattle, dogs and cats with progressive neuromuscular dysfunctions; missense and nonsense mutations	000402–9940; 000402–9685; 000402–9615;	ENSECAG 00000011942	16:51.36–51.37	12	Loss	Fell Pony	This study
*KRT1*	*IMMUNE*: Epidermolytic hyperkeratosis in dogs; missense mutation	001415–9615	ENSECAG 00000022233	6:69.75–69.77	21	Loss	Akhal-Teke, Belgian, Fell Pony, Friesian, Mongolian, Standardbred, Swiss Warmblood	This study, [37], [39]

### Composite CNV dataset for the horse genome

**Figure 4:**
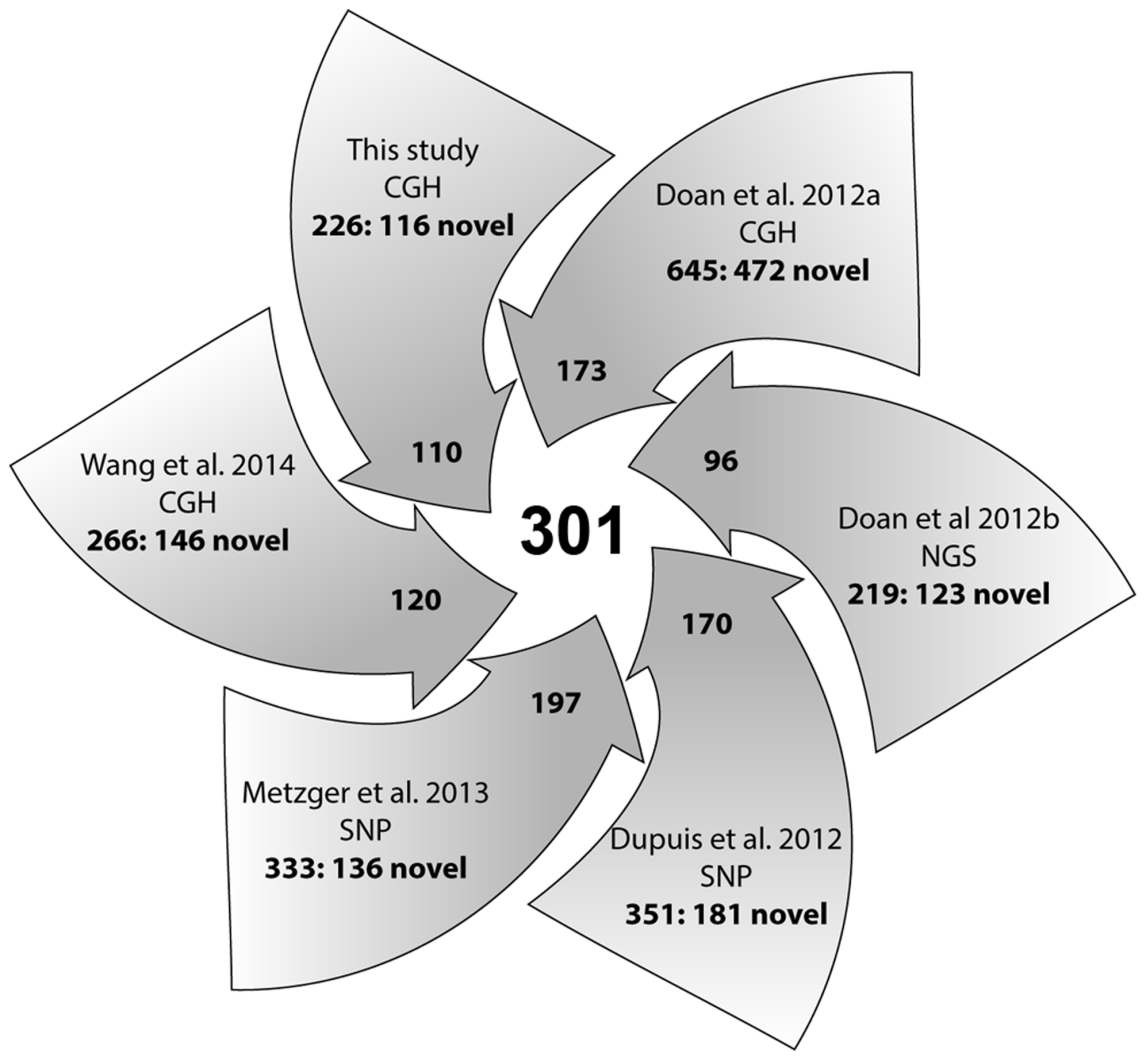
A summary diagram of all CNV studies in horses and their contribution to the integrated CNV dataset. Numbers in arrow-heads denote the contribution of each study to the common pool of **301** shared CNVRs; numbers in arrow-tails denote the total and novel (separated by colon) CNVRs per study; CGH, SNP and NGS denote the platforms used for CNV detection.

Comprehensive knowledge of CNVs in normal horse populations, within and across breeds, is a prerequisite for the discovery of variants that contribute to equine genetic diseases and disorders. Therefore, we aligned the 258 CNVRs identified in this study with previously published CNV data for the horse [35], [36], [37], [38], [39]. Altogether, we found records of about 2041 CNVs and CNVRs (calling criteria vary between studies). These were further consolidated, based on adjacent locations or partial overlaps, into 1476 CNVRs of which 301 CNVRs (20%) were shared between two or more studies (Table S10, Fig. 4). The majority of shared CNVRs involved genes associated with olfactory reception (50 CNVRs) and membrane transport (49 CNVRs) but also genes involved in transcription (30 CNVRs), cell cycle regulation (12 CNVRs) and RNA genes (34 CNVRs). Expectedly, CNVRs that were found in more than 100 horses and reported by all 6 studies exclusively involved olfactory receptors. Comparative analysis also revealed that novel (study-specific) CNVRs predominated over shared ones in all 6 studies (Fig. 4). Novel CNVRs of functional interest from this study involved genes related to sperm-egg interaction and fertilization in chr4:19.8–19.9 Mb; a developmental gene *SOX2* in chr19:20.1 Mb; an X-linked region harboring genes of circadian pacemaker function chrX:83.8–84.0 Mb, and a complex CNVR in chrUn:225–226 kb with cancer related genes. Notably, the latter two CNVRs were found in more than 10 horses each. Details of all novel and shared CNVRs are presented in Table S10.

### Experimental validation of CNVRs by quantitative PCR and FISH

**Figure 5:**
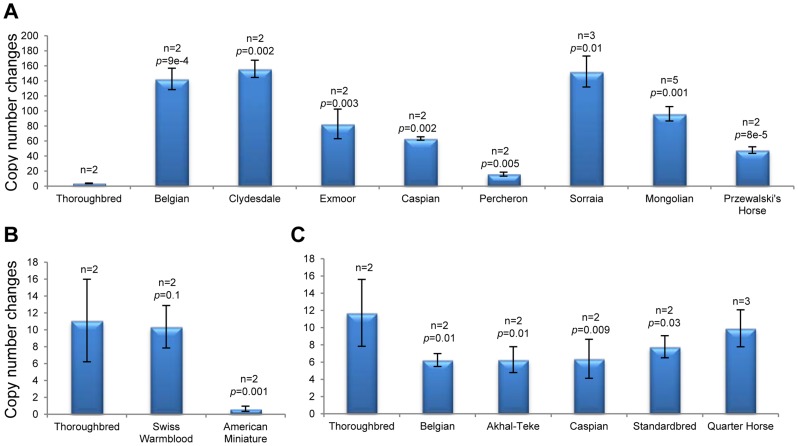
Validation of CNVRs by PCR. **A**. gains and **B**. losses in Chr27 (37.3 Mb; probe Gs_27_37371896) involving CUB and Sushi multiple domains 1 (*CSMD1*) gene; **C**. Loss in Chr20 (24.8 Mb; probe Eic_20_24841849) involving olfactory receptors; n – number of individuals analyzed.

Nineteen CNVRs were validated by quantitative PCR (qPCR) using array probe-specific primers (Table S2). The regions were selected upon three criteria – size, gene content and novelty. The smallest tested CNVR was 4 kb and the largest 2 Mb; 13 involved clusters of horse genes, and 6 were novel. A summary of qPCR results are presented in Figure S3 and Table S11. All selected CNVRs were first tested in the discovery horses and then analyzed in more individuals of the same breed to identify possible breed-specific tendencies. Overall, qPCR observations agreed well (*P*-value <0.05) with the array CGH data for all discovery horses and for other animals of the same breed. For example, it confirmed a complex CNVR in chr27 involving *CSMD1* gene (CUB and Sushi multiple domains 1) which encodes a transmembrane and a candidate tumor suppressor protein [43]. Copy numbers in this region were tested on 11 breeds with at least 2 individuals each and showed a gain in native ponies, draft breeds and the Przewalski horse, and a loss in American Miniature horses in relation to the Thoroughbred (Fig. 5A–B). Likewise, qPCR confirmed a CNVR in chr20 (Fig. 5C) which has been found only in this study and in indigenous plateau horses [38]. However, we found some differences too between the two data sets: e.g., while qPCR confirmed a loss in chr20:32.0–32.4 Mb and chr17:18.8–19.0 Mb in the discovery Swiss Warmblood and Mongolian horses (Table S3), respectively, inclusion of additional horses from the same breeds resulted in a significant gain in these regions (Fig. S3). Also, initial qPCR confirmed a loss in chr7:74.8–74.9 Mb in the two discovery Swiss Warmblood horses (Table S3) but no significant losses were found when more individuals were added. These minor discrepancies can be attributed to intra-breed variation: array CGH was based on 2 to 4 individuals, while qPCR involved 4 or more horses per breed (Figure S3, Table S11).

**Figure 6:**
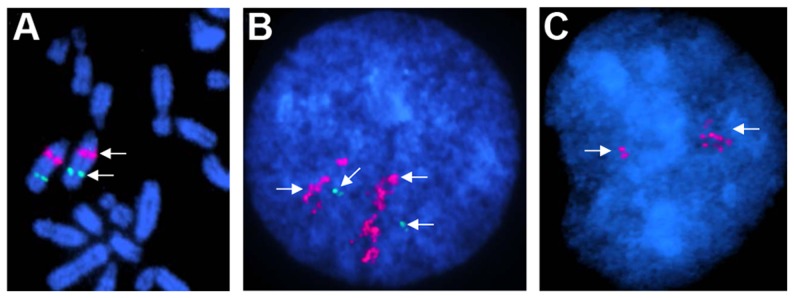
Chromosomal assignment and validation of a CNVR in chrUn (529–531 kb) by FISH. **A**. Mapping the CNVR to chr19q12–q13 by FISH with BAC 132B13 (red); green - a control BAC with *UMPS* gene in chr19q21 [93]; **B**. The CNVR (red) in interphase chromosomes of a Percheron; green – a single-copy control probe; **C**. The CNVR (red) in interphase chromosomes of a Thoroughbred (*Twilight*). Note the difference in copy numbers between the Percheron and the Thoroughbred, as well as between homologous chromosomes.

Two CNVRs, a complex 200 kb gain-loss region in chr1:114.0–114.2 Mb and a 2.2 kb gain in chrUn: 529–531 kb) were validated by FISH using CNV-containing CHORI-241 BAC clones 132B13 (Fig. S4) and 91B23 (Fig. 6), respectively. Clear differences in copy numbers between individual horses, as well as between homologous chromosomes of the same horse were observed. Additionally, the CNVR in chrUn was mapped to horse chr19q12–q13 (Fig. 6).

### Discovery of CNVs in horses with XY disorders of sexual development (DSDs)

Finally, we carried out a pilot study to test the utility of the tiling array and the integrated CNV data set (Table S10) for the discovery of CNVs involved in equine XY disorders of sexual development (XY DSD). Selection of the phenotype was based upon studies in humans suggesting contribution of CNVs to XY DSDs [25], [27], [28]. Array CGH experiments were carried out in 6 affected horses (Table 4): all had normal male 64,XY karyotype with an intact *SRY* gene, abnormal male or female gonads, and female or female-like external phenotype [44]. We determined 179 CNVs (average 30 calls per individual) and 107 CNVRs, of which 83 were common and shared with normal equine populations, and 24 CNVRs were novel (Table 5). Only 3 novel CNVRs were shared between two or three XY DSD horses, while the remaining 21 were private and present in just one animal. Protein coding or miRNA genes with functions in cell cycle regulation, transcription and posttranscriptional processing were involved in 14 novel CNVRs. None of the CNV-genes had known functions in sexual differentiation or development.

**Table 4:** Horses with *SRY*-positive XY DSDs analyzed in this study.

Horse ID	Breed	Phenotype	Karyotype	*SRY* PCR	Reference
H169	Appaloosa	Normal external genitalia; hypoplastic uterus; underdeveloped mammary glands; estrous behavior	64,XY	pos	[44]
H252	Mixed warmblood	Small ventrally located vulva between rear legs; abnormally small uterus	64,XY	pos	[44]
H348	Standardbred, *Arizona Helen*	Female-like external phenotype; rudimentary abdominal gonads (testes) - male pseudohermaphrodite	64,XY	pos	[44]
H369	Standardbred *Martha Maxine*	Female-like external phenotype; rudimentary abdominal gonads (testes) - male pseudohermaphrodite	64,XY	pos	[44]
H544	Tennessee Walking Horse	Female-like external phenotype; rudimentary abdominal gonads (testes) - male pseudohermaphrodite	64,XY	pos	This study
H546	Thoroughbred	Female-like external phenotype; rudimentary abdominal gonads (testes) - male pseudohermaphrodite	64,XY	pos	This study

**Table 5:** Novel and highly aberrant common (in bold font) CNVRs in XY DSD horses.

Horse ID	Chr	Start	Stop	Size, kb	Gene symbol or sequence ID	log_2 average_
H169	3	57,377,730	57,394,356	16.6	Intergenic	−0.7
H546	4	95,018,254	95,034,834	16.5	ENSECAG00000014506	−1.0
H369	8	13,128,936	13,134,708	5.7	*MZT2B, TUBA3D*	−1.0
H544	10	51,078,681	51,119,659	40.9	Intergenic	−0.9
H545	12	619,419	638,513	19.0	*CSTF3*	−0.7
H544, H546	14	15,353,340	15,560,610	207.2	ENSECAG00000002162	−0.7
H544	14	29,406,214	29,432,771	26.5	*TET2*	−1.3
H369	15	14,237,934	14,272,519	34.5	*BCL2L11*	−0.7
H544	15	44,348,596	44,377,735	29.1	eca-mir-217, eca-mir-216a, eca-mir-216b	−1.1
H369	16	21,651,711	21,683,467	31.7	Intergenic	−0.9
H546	16	65,654,753	65,661,154	6.4	Intergenic	−1.1
H544	18	14,064,543	14,101,261	36.7	JU909423	−1.0
H546	18	60,925,026	60,954,644	29.6	ENSECAG00000003850	−1.2
H252	20	18,917,213	18,969,026	51.8	*E2F3*	+0.8
H546	20	36,503,850	36,508,477	4.6	*SRSF3*	−1.0
H546	23	27,814,249	27,838,233	23.9	*GLDC*	+0.8
H544	23	46,897,740	46,957,535	59.7	AK140548	−0.9
H369	24	44,255,536	44,269,279	13.7	Intergenic	−0.7
H544	25	22,614,255	22,768,706	154.4	JO239254	−0.5
H544	26	5,796,076	5,840,516	44.4	*TIGD1*	−1.2
H252	27	20,201,870	20,232,375	30.5	*MICU3*	−0.5
H369, H544, H546	28	18,833,995	18,846,757	12.7	*UBE2N*	−1.1
H252	X	203	366,729	366.5	*AKAP17A, ASMT, ZBED1, XG*	−0.5
H544, H546	X	98,506,468	98,543,836	37.3	*STAG2*	−1.1
**H252**	**7**	**74,885,505**	**74,911,413**	**25.9**	*OR56B4*	−2.2
**H348, H369**	**29**	**28,640,862**	**28,835,337**	**194.4**	*AKR1CL1, AKR1C2, AKR1C3, AKR1C4*	−3.5

**Figure 7:**
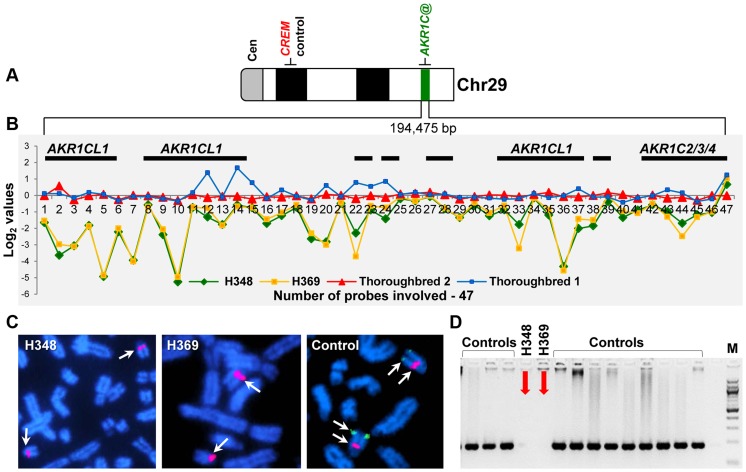
Schematic of the homozygous deletion in chr29, 28.6–28.8 Mb in two XY DSD horses. **A**. chr29 ideogram showing the location of *AKR1C* genes and a control gene *CREM*; **B**. Detailed map of the CNVR showing the location of genes (black horizontal bars) and CGH signal log2 values for 47 array probes in XY DSD and control horses; **C**. FISH results with a BAC 23N13 spanning the deletion (green signal) and a control BAC 76H13 for *CREM* from a non-CNVR (red signal); **D**. PCR with CNVR-specific primers in XY DSD and control horses.

Analysis of common CNVRs for highly aberrant log_2_ values detected two likely homozygous deletions (Table 5): a 26 kb loss in chr7 (log_2_ −2.2) and a ∼200 kb loss in chr29 (log_2_ −3.5). The latter was of particular interest because it was found in two closely related American Standardbreds with very similar male-pseudohermaphrodite phenotypes (H348 and H369; Table 4). The CNVR was also present in 10 out of the 38 normal horses (Table S3) including one American Standardbred, though with a moderate aberration value (log_2average_ −0.7) compared to log_2_ = −3.5 in the two XY DSD horses. Most notably, the CNVR involved at least 4 members of the aldo-keto reductase *AKR1C* gene family, known to be critical in the backdoor pathway of dihydrotestosterone (DHT) synthesis and sexual development [45], [46]. A schematic overview of the CNVR, including the involved genes and aberration profiles of all 47 array probes in the region, is presented in Fig. 7. Homozygosity of the deletion was confirmed by fluorescence *in situ* hybridization (FISH) with a BAC clone (CHORI-241-23N13) spanning the deletion. The BAC hybridized to chr29 in control animals but not in the two XY DSD horses, whereas a control BAC (CHORI-241-76H613) with the *CREM* gene from a non-CNVR in chr29 [47] hybridized equally in the XY DSD horses and controls (Fig. 7). Homozygosity of the deletion was further confirmed by PCR showing that primers designed inside the CNVR amplified genomic DNA of control horses and the remaining 4 XY DSD horses, but not of the two male-pseudohermaphrodite American Standardbreds (H348 and H369; Fig. 7). Though primers designed outside the CNVR, amplified the DNA by PCR in all horses – an evidence that the DNA quality of the two Standardbreds was acceptable. We theorized that the homozygous deletion involving *AKR1C* genes in the two male-pseudohermaphrodite horses might be the risk factor for abnormal sexual development.

## Discussion

During just the past two years, five studies have addressed the phenomenon of copy number variation in the horse genome [35], [36], [37], [38], [39] contributing to our knowledge about the genomic landscape of CNVs and their role in inter-individual variation in horses. Despite the progress, lessons from humans [48], [49], [50] and more recently from dogs [7], [34], show that efficient biomedical application of this information requires integration of data from many more populations and individuals and the use of comparable methodological platforms[48], [49], [50].

Here we report about the construction of a 400K high-density WG tiling oligoarray for the horse and its application for the discovery of CNVs in 38 normal horses of 16 diverse breeds, as well as in 6 horses with congenital disorders. Probes on the array were designed to detect CNVs in 18,763 equine autosomal and X-linked genes but also in intergenic, sub-telomeric and Y chromosome sequences. Regarding genome coverage, our CNV discovery platform most closely resembled the recently reported WG 1.3 M NimbleGen CGH array [38], but essentially complemented the exon CGH array by Doan and colleagues [39] and the studies based on WG SNP50 BeadChip [37], [51]. The latter is of a magnitude lower density and not specifically designed for CNV capture. Also, as shown in humans and cattle, the efficiency of CNV discovery is lower in SNP platforms compared to CNV focused arrays [29], [50]. While the future direction for CNV research in any species is probably next generation sequencing (NGS), the approach has as yet found only limited application in horses: for the discovery of CNVs in the genome of a Quarter Horse mare [35] and for the discovery of segmental duplications in 6 horse breeds and the donkey [52].

A unique feature of our CGH array was the inclusion of probes from the Y chromosome and sub-telomeric regions. This was because CNVs and segmental duplications are known to be an integral part of the architecture of the mammalian Y chromosome [53], [54], while sub-telomeres are hotspots of DNA breakage and repair, and undergo structural rearrangements more frequently than the rest of the genome [40], [55]. Despite the almost 6,000 sub-telomeric probes with lower than average spacing (∼4 kb *vs.* ∼7 kb across the genome) on the array, only 10 CNVs were detected in sub-telomeres and none in the Y chromosome (Table 2). It is likely that the complex sub-telomeric sequences are either missing or underrepresented in the current horse sequence assembly [56], due to which it is possible that the probes designed from the ends of the chromosomes, did not originate from actual sub-telomeres.

Poor representation of centromeric/pericentromeric and telomeric/sub-telomeric sequences is a common shortcoming of all draft genome assemblies. Whilst the horse may be different to humans or other species in terms of subtelomeric sequences, this can only be rigorously shown by sequencing BAC clones from these regions, preferably with long-read single molecule technology such as a Pacific Biosciences instrument to resolve long repeats. Such an approach was recently successfully applied to resolve regions of segmental duplications in the finished genome sequence of humans [57].

The Y chromosome, on the other hand, has acquired and amplified novel sequences, as well as sequences from the rest of the genome [58]. Thus, it is likely that many potential copy number variable Y probes did not pass the ‘uniqueness’ test by BLAST and were dropped from the array (see Material and Methods for details).

### Integration and comparison of the CNV data for the horse

The present and all previous CNV studies in horses [35], [36], [37], [38], [39] differ by discovery platforms, genome coverage, resolution, the study cohorts and analytical methods (Table 6). Therefore, the overall numbers, size ranges and chromosomal distribution of CNVs vary between the studies. For example, it has been shown that due to analytical reasons, CGH-based studies tend to detect more losses than gains [59]. This holds true for the Agilent WG array in the present study and also the Nimblegen WG array [38], though [38]slightly more gains were detected with the Agilent exon array [39] (Table 6). The latter was attributed to the large number of losses in the reference animal compared to the Thoroughbred (*Twilight*) genome sequence assembly EquCab2 [56]. In contrast, gains vastly predominate (97%) among the CNVs found by NGS in a Quarter Horse mare [35]. Apparent differences in CNV calling algorithms and thresholds (Table 6), on the other hand, are responsible for the variation in the number of CNVs, their size and the criteria for merging individual CNVs into CNVRs. For example, in this study we mainly reported CNVRs because this is how the ADM-2 algorithm analyses and assembles the CNV calls (CNVs) within and across individuals. Further, specific features of the probe/array design, and not necessarily the number of probes, are responsible for the differences in the genomic distribution of discovered CNVs. So far, X-linked CNVs have been found only in this study and by Doan & colleagues [39], and CNVs in chrUn only in this study. Surprisingly, the study with a three times denser 1.3 M Nimblegen array failed to detect CNVs in chrX, as well as in [38] chrs30 and 31 [38]. At the same time, the latter two small autosomes show the highest number of CNVs in the Quarter Horse mare [35]. Major differences are also in the size, diversity and origin of the study cohorts, ranging from just a few breeds and individuals [35], [38] to over 15 breeds (this study and [37]) and hundreds of individuals [36], [37] (Table 6).

**Table 6:** Summary statistics of all CNV studies in horses.

	This study	Doan *et al.* 2012a	Doan *et al.* 2012b	Dupuis *et al.* 2012	Metzger *et al.* 2013	Wang et al. 2014
**Platform**	Tiling array	Tiling array	n/a	SNP Beadchip	SNP Beadchip	Tiling array
**Production company**	Agilent	Agilent	n/a	Illumina	Illumina	Nimblegen
**Genome coverage**	WG	Exons and UTRs	WG	WG	WG	WG
**No of probes**	400K	400K	n/a	50K	50K	1.3 M
**Method**	CGH	CGH	NGS	SNP genotyping	SNP genotyping	CGH
**Breeds**	16	15	1	4**	17	6
**Horses**	38	16	1	477	717	6
**CNV calling algorithm**	ADM-2	ADM-2	Control-FREE copy number (FREEC)	PennCNV [99]	1) CNVPartition (Illumina); 2) PennCNV [99]; 3) QuantiSNP [100]	segMNT
**CNV calling threshold**	Log_2_±0.5, 5 probes	Log_2_±0.5, 3 probes	Breakpoint at −0.0013; a coefficient of variation 0.045	PennCNV [99]	***	Log_2_±0.5, 5 probes
**No of CNVs per animal**	12 to 59	55 to 347	282	n/a	Min 1, max n/a	22 to 84
**No of CNVs/CNVRs** *	258	775	282	478	166–1090	353
**Gains**	64	398	274	238	n/a	109
**Losses**	172	315	8	236	n/a	234
**Complex**	22	62	n/a	n/a	n/a	n/a
**CNV size range**	1 kb–2.5 Mb	197 bp–3.5 Mb	3.7 kb–4.8 Mb	97 bp–2.7 Mb	516 bp–0.9 Mb**	6.1 kb–0.5 Mb
**CNV size, mean, kb**	110	5.3	n/a	114	487	38.5
**CNV size, median, kb**	46	99.4	n/a	61	169	13.1
**Genomic distribution of CNVs**	Autosomes, X, Un	Autosomes, X	Autosomes	Autosomes	Autosomes	Autosomes, except chr30, 31
**Most enriched chr.**	12	12	12	12	12	12
**Chrs. with the highest no. of CNVs**	1; 20	1; 7	30; 31	1	12	20
**Genome enrichment %**	1.15	3.65	3.53	2.32	1.7–22.0**	0.61

*As reported by original studies and before consolidating overlapping and tandemly located CNVRs into a composite dataset.

**Dupuis and colleagues specified only large groups of horses (warmblood, coldblood, draft, pony) but not individual breeds.

***Results by Metzger and colleagues vary between different analysis software packages used.

The many variables between the six studies (Table 6) obviously confound assessments based solely on CNV metrics, and it would probably be more appropriate to compare the actual CNVs/CNVRs reported. Therefore, and in order to obtain a comprehensive overview about the status of CNV discovery in horses, we integrated the CNVs or CNVRs from all six studies ([35], [36], [37], [38], [39], this study) according to their genomic locations into a composite dataset of 1476 CNVRs (Table S10). Of these, 301 are reported by at least two studies, while the remaining 1174 CNVRs are study-specific (novel; Fig. 4) and require further validation.

The integrated dataset is a needed resource for evaluating new CNV discoveries and gives an idea about the most intrinsic features of the CNV profile in horses. Copy number variants account for about 1 to 3 % of the horse genome and there are more CNVs that involve genes than those located in intergenic regions. Though, the number of intergenic CNVs is possibly deflated because all tiling arrays [38], [39], including ours, have been biased towards probes for gene exons. For example, 20% of the probes in the Texas-Adelaide WG array represent protein coding genes, whereas these genes make up only about 2–3% of the mammalian genome. Notably, all studies find chr12 as the most CNV-enriched (Table 6) and not because of many CNVs, but because of a few very large clusters of olfactory receptors and immunity-related genes (Tables S8, S10).

### Copy number variants and segmental duplications

Studies in human [3], [60], dogs [8] and cattle [30] have noted strong correlation between CNVs and segmental duplications (SDs). This is because SDs share 90% sequence similarity with another genomic location and can promote CNV formation by non-allelic homologous recombination [61]. Similar tendency has been observed in horses [39], although horse SDs are relatively small (largest ∼60 kb) and comprise only about 0.5–0.6 % of the genome [56], thus less than the portion involved in CNVs (Table 6). Low level of SDs or low copy number repeats was also reported by a recent *de novo* analysis of the equine genome where no novel classes or types of interspersed repeats were identified [62]. An additional 0.4% of SDs are in unplaced contigs (chrUn) [56], though in this study only 0.04 % of chrUn sequences had CNVs (Table 2). Likewise, chr25 which is the most SD-rich chromosome (1.7%) according to EquCab2 genome assembly [56], was only moderately enriched with CNVs (0.35%) in this study. Yet, findings by us and others support the correlation between CNVs and SDs in some genomic regions. For example, a known large (750 kb) segmental duplication at the boundary of ELA class I and class III [63] falls into a large common CNVR in chr20:30,127,886–31,231,182 (Table S10); further, low copy number directional repeats have been associated with large deletions in the horse Y chromosome [44] or, GO categories, such as olfactory reception and immune response, prevail among the genes involved both in CNVs and SDs [52]. Therefore, for improved understanding of the genomic architecture of CNVs and their relation to genes and phenotypes in horses, it would be worthwhile to focus future CNV research on associations between CNVs and SDs, as recently successfully done in dogs [8].

### Copy number variable genes and intergenic regions

It is noteworthy that regardless of the discovery methodology and study cohorts, functional groups of genes that are most affected by CNVs remain the same in all studies. These include genes for transmembrane signal transduction and chemo-attractant sensory perception (olfactory and non-olfactory G-protein coupled receptors, GPCRs), immune response (immunoglobulins, T-cell receptors, MHC protein complexes), and steroid metabolism (Table S9). Not coincidentally, CNVs are associated with the same groups of genes in humans [3], [64], cattle/ruminants [30], [65], [66], pigs [31], dogs [32] and even chicken [67], suggesting the importance of inter-individual variation in these genes for adaptive plasticity [68]. Indeed, genetic diversity and fine functional tuning of sensory receptors, immunoglobulins, natural killer and Toll-like receptors is further enhanced by additional mechanisms, such as asynchronous replication which increases the rate of tandem duplications, and monoallelic expression, so that each sensory neuron or lymphoid cell expresses only one allele of a gene [69], [70]. Conserved linkage between distinct olfactory receptor genes and the MHC in several mammalian species suggests their concerted function - in this case, MHC-influenced mate choice in reproduction [71]. Olfactory receptors are also thought to function as chemo-sensing receptors to regulate sperm density, motility, acrosome reaction and sperm-egg interaction in fertilization [71], [72]. Thus functionally, the CNV-enriched genes in horses and other mammals fall into just three large categories: sensory perception, immunity and reproduction.

Among the 258 CNVRs detected in this study, 20% were located in intergenic regions. These CNVRs were relatively small (average 50 kb, median 35 kb) and represented predominantly losses (Fig. 2, Table S8). Prevalence of losses among intergenic CNVRs has also been found in dogs [32]. Although there is no information about possible implication of these regions on the function of genes in animal genomes, studies in humans show that intergenic deletions are significantly enriched among gene expression-associated CNVs [73]. Thus, with the improvement of genome sequence assembly and annotation in horses, intergenic CNVRs will be of interest for future studies. We also anticipate that as gene models are revised and converge more with the underlying reality of the genes, some intergenic CNVRs may become genic and *vice versa*.

### Breed-specific CNVs

**Figure 8:**
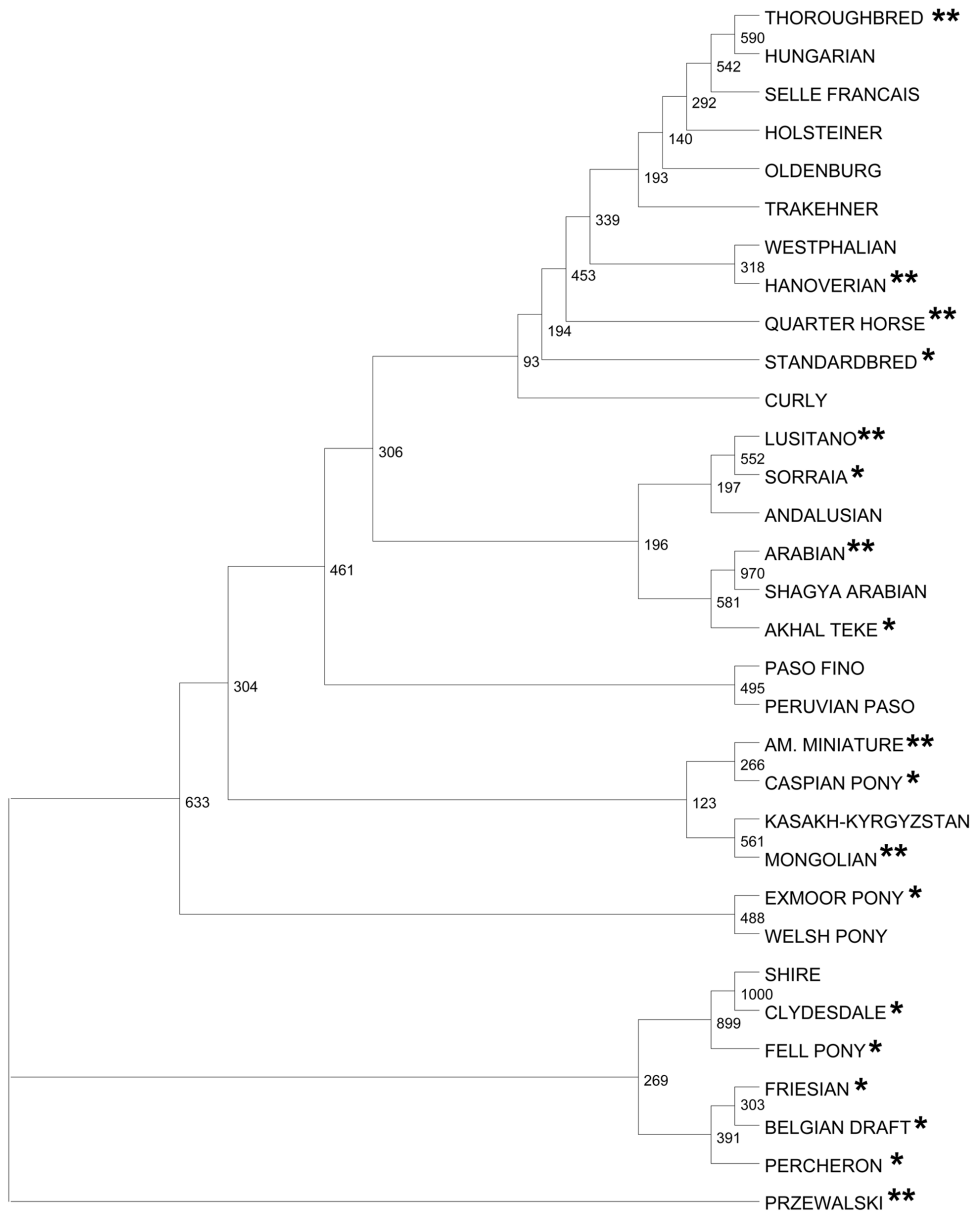
Genetic relationships of horse breeds studied for CNVs. A Maximum Likelihood tree showing genetic relationships of the horse breeds that have been studied for CNVs; * new breeds added in this study (except Swiss Warmblood);** breeds involved in 2 or more studies. Numbers denote bootstrap values.

One of the goals of CNV research in horses is to find variants that distinguish between breeds or groups of breeds and could be associated with specific adaptations and phenotypic traits of interest. In order to visualize the breeds and the degree of diversity represented in this and previous studies, we performed a phylogenetic analysis using population data of 15 microsatellite loci [74] for the breeds involved (E.G. Cothran, unpublished). The dendrogram in Figure 8 shows that while the major clades of domestic horses are represented, there is a clear preponderance of the breeds with Thoroughbred ancestry. It is therefore noteworthy that data for 11 new breeds, mainly representing native ponies and draft horses, were added in this study. Nevertheless, the current tally of horse breeds studied for CNVs is 41 (Table S12) which is less than 10% of the over 400 horse breeds known worldwide [75]. Furthermore, given that just 7 breeds have been involved in 2 or more studies (Fig. 8, Table S12) and several breeds are represented by one individual [38], [39], any CNV reported to be breed-specific should be taken with caution. For example, our composite CNV dataset (Table S10) shows that the 18 CNVs reported to be specific for Hanoverians [37] are present in other breeds. Likewise, only one (chr13: 1,497,390.00–1,508,926.00; *EIF2AK1*) of the 7 plateau-breed-specific CNVs in heme binding genes [38] is not found in other breeds. The same happened with our data where initially we identified over 10 putative breed-specific CNVs which, after comparison, reduced to 2 - one in Exmoor pony, another in Swiss Warmblood horse (Table S4). Interestingly, no unique CNVs were found in the Przewalski horse which shared similarity mainly with ponies and draft breeds (Table S3). Besides, only 9 of the 25 CNVs in Przewalski horses were shared between the two individuals studied. Similar tendency for intra-breed individual variation was observed for domestic horses where private CNVs predominated over the shared ones. Nevertheless, as suggested by other studies in horses [39], cattle [29], pigs [31] and dogs [33],we anticipate that a small percentage of CNVs might remain unique to their respective breeds, though this requires analysis of much larger and more diverse equine populations. On the other hand, most horse breeds are of recent origin with a good deal of cross-breeding until closed breeds were established which has led to a high degree of haplotype sharing [56], [76], and thereby decreased chances for finding breed-specific CNVRs compared to species like dogs [34].

### CNVs and disorders of sexual development

Probably the most exciting goal of CNV research in any species is the discovery of pathogenic variants responsible for complex diseases and congenital disorders. Among these, disorders of sexual development (DSDs) are not uncommon in horses, though causative mutations have been identified for just a few: Y chromosome deletions in *SRY*-negative XY sex reversal mares [44] and a point mutation in the androgen receptor gene in 3 related *SRY*-positive XY mares [77].

Here, we conducted the first pilot CNV analysis in horses with XY DSD and identified a large autosomal (chr29) deletion in 2 related American Standardbreds (H348 and H369, Table 4). The animals were classified as male pseudo-hermaphrodites with XY male genotype, immature testes-like abdominal gonads, and female-like external phenotype (Table 4). The deletion in chr29:28.6–28.8 Mb was homozygous as confirmed by FISH and PCR, and involved at least 8 genes of which 4 belonged to the aldo-keto reductase family 1, member C (*AKR1C*; Fig. 7). Annotation of these genes in the equine genome is, as yet, preliminary and based on the alignment with human AKR1C proteins in the UCSC Genome Browser (http://genome.ucsc.edu/index.html) and mammalian homology in Ensembl (http://www.ensembl.org/index.html). Therefore in Fig. 7, three genes are denoted as *AKR1CL1* and one gene has three labels, corresponding to *AKR1C2* in chimpanzee, *AKR1C3* in human, and *AKR1C4* in cattle.

The *AKR1C* genes are members of the aldo-keto reductases (AKR) superfamily [78]and encode for 3α-hydroxysteroid dehydrogenases [78] which are critically involved in steroid hormone metabolism [79]. In the human genome, there are 4 family members - *AKR1C1, ALR1C2, AKR1C3* and *AKR1C*4, which share 86% sequence identity and are clustered in HSA10p15-p14 [78], [79]. The human *AKR1C* genes are not widely expressed: *AKR1C1* in brain, kidney, liver and testis, *AKR1C2* in prostate and brain, *AKR1C3* in prostate and mammary gland, and *AKR1C4* in liver, whereas the rat has a single *AKR1C* gene expressed in liver[79], [80], [81]. Among other functions, the *AKR1C* genes are involved in the biochemical pathway that leads to dihydrotestosterone (DHT) synthesis without testosterone intermediate. As opposed to ‘classical’ DHT synthesis from cholesterol and testosterone, this pathway is known as ‘the backdoor pathway’ and was originally discovered in marsupials [82] and thereafter in eutherian mammals [45], [46], [83], [84]. The importance of the ‘backdoor pathway’ and *AKR1C* genes in male sexual development was recently demonstrated by a study in humans showing that mutations in *AKR1C2* and *AKR1C4* genes cause abnormal virilization and disordered sexual development, including XY sex reversal [46], [84]. Even though no mouse knockout models are available for any of the *AKR1C* genes (MGI; http://www.informatics.jax.org/), it is tempting to speculate that the homozygous deletion in horse chr29 is a causative or a risk factor for some forms of equine XY DSDs, such as male-pseudohermaphroditism, as observed in this study. It is also worth mentioning that a CNV analysis of human XY DSDs detected a clinically significant *de novo* 64 kb duplication in HSA10p14 [28] - a genomic segment next to the *AKR1C* gene cluster (UCSC: http://genome.ucsc.edu/cgi-bin/hgGateway). Whether this is a coincidence or the region includes more copy number variable factors contributing to DSDs, needs further investigation. [45], [46], [84] [84] [28].

Our findings in horses might be of even broader interest because the two deletion carrying horses were elite American Standardbred pacers, *Martha Maxine* and *Arizona Helen* (Table 4), whose problematic sexual identity has become public, making headlines in *The New York Times* [85] and *The Horse* [86]. Thus, studies are underway to precisely determine the deletion breakpoints and develop molecular tests for detecting other horses with a similar deletion, as well as heterozygous carriers. Finally, the fact that only 2 XY DSD horses out of 6 had this mutation underscores the phenotypic and genetic heterogeneity of these disorders.

### Concluding statement

This study represents an important contribution to CNV research in horses by identifying new CNVs and developing an integrated datset of 1476 CNVRs to facilitate the discovery of variants of biomedical importance. However, despite progress, the majority of the CNVRs reported for the horse require proper validation by methodologically comparable studies invloving more diverse breeds and individual animals. Last but not least, due to the very nature of CNVs, these regions are likely to have sequence assemblies not as accurate as non-variable regions. Thus, the findings also identified potential targets for genome re-sequencing and -assembly.

## Materials and Methods

### Ethics statement

Procurement of peripheral blood and hair was performed according to the United States Government Principles for the Utilization and Care of Vertebrate Animals Used in Testing, Research and Training. These protocols were approved by Texas A&M Office of Research Compliance and Biosafety as AUP2009-115, AUP2012-0250. CRRC09-32 and CRRC09-47.

### Array design

A horse WG tiling array was designed using the horse genome draft sequence (EquCab2, http://www.ncbi.nlm.nih.gov/assembly/286598; [56], Oligowiz2.0 (http://www.cbs. dtu.dk/services/OligoWiz/), ArrayOligoSelector (http://arrayoligosel.sourceforge.net/), and ArrayDesign [87] software packages. The array comprised 417,377 60-mer oligonucleotide probes: 85,852 probes corresponded to one or more exons of the 18,763 annotated equine genes (http://www.ncbi.nlm.nih.gov/genome/genomes/145?); 305,416 probes originated from intergenic regions (excluding sub-telomeres); 5,716 probes were designed from sub-telomeres (the terminal 1 Mb of each chromosome), and 519 probes represented the horse Y chromosome [58]; our unpublished data). [87]For intergenic probes, including chrUn, repeat-masked (http://www.repeatmasker.org/) sequences were used. For reference genes, we first designed probes from exons. If these were not specific, attempts were made to design probes from introns and upstream/downstream flanking regions of those genes. Before inclusion in the array, the specificity of all sequences were analysed with BLAT (http://www.kentinformatics.com/) and BLAST (http://blast.ncbi.nlm.nih.gov/Blast.cgi) against the EquCab2 reference genome sequence. Probes with more than one hit in the genome were discarded. Possible cross-hybridization of the probes was further evaluated using Kane's parameters [88] and all probes that had a total percent identity >75–80% with a non-target sequence, or probes with contiguous stretches of identity >15 nucleotides with a non-target sequence were discarded. Only probes with high specificity were kept in the final array. A Cytoband file was generated to align the horse draft sequence assembly with the cytogenetic map [89]. The array, designated as the Texas-Adelaide horse WG tiling array, was fabricated by Agilent Technologies using Agilent SurePrint G3 technology and 2×400K chip format (two arrays on a single slide). The array is available at Agilent Technologies; Design ID #030025, Cat. No G4124A.

### Horses, breeds, phenotypes

The CNV discovery cohort comprised 38 horses representing 16 diverse breeds and the Przewalski's horse (Table S1). Horse breeds were selected according to the recent population studies [51], [56], [76], [90] with an aim to maximize the genetic diversity among samples and to encompass the common warm blood, cold blood (draft) and native pony breeds. An additional cohort of 52 normal horses representing the same 16 breeds was used for quantitative PCR validation of CNVs. Finally, a pilot study testing the utility of the tiling array for the discovery of CNVs contributing to equine congenital disorders used 6 horses previously diagnosed with XY disorders of sexual development (XY DSDs; Table 4) [44].

### DNA isolation

Genomic DNA was isolated from peripheral blood or hair follicles using QIAGEN Gentra PureGene Blood kit (Qiagen) according to manufacturer's protocol. The DNA was cleaned with DNeasy Blood and Tissue kit (Qiagen) and quality checked by gel electrophoresis and by Nanodrop spectrophotometry (Thermo Scientific).

### Array comparative genomic hybridization

Probe labeling and array CGH experiments were performed according to Agilent Technologies Protocol Version 7.3, March 2014 (http://www.chem.agilent.com/Library/usermanuals/Public/G4410-90010_CGH_Enzymatic_7.3.pdf). All hybridizations comprised of a pair of differently labeled probes, one of which was always the reference DNA – a Thoroughbred mare *Twilight* for females and a Thoroughbred stallion *Bravo* for males (see explanations below). The genomic DNA (gDNA) was cleaved to 200–500 bp fragments with *RsaI* and *AluI* (Promega) and labeled with Cy3 (the reference DNA) or Cy5 (sample DNA) by random priming using Genomic DNA Enzymatic Labeling Kit (Agilent Technologies). The products were cleaned with 30 kDa filters (Amicon) and the yield and specific activity of labeled DNA was determined with a Nanodrop spectrophotometer. Typical yield for 1 µg of starting DNA was 6–8 µg; specific activity for Cy3 was 25–40 pmol/µg and for Cy5 20–35 pmol/µg. The hybridization mixture was prepared using Agilent Oligo aCGH Hybridization Kit and contained equal quantity of Cy3 and Cy5 labeled probes, 1 µg/µL horse Cot1 DNA, 10× blocking agent, and 2× Hi-RPM buffer. Denatured and pre-annealed probe mixture was applied onto gasket slide, placed in Agilent SureHyb hybridization chamber, ‘sandwiched’ with an array slide and incubated in Agilent hybridization oven at 65°C for 40 hours. The array slides were washed with Agilent aCGH Wash Buffers 1 and 2 and dried with Acetonitrile and Stabilization and Drying Solutions (Agilent Technologies).

### Array CGH data analysis

The slides were scanned with Agilent SureScan DNA Microarray Scanner and Scanner Control software v8.3. The data were extracted and normalized with Agilent Feature Extraction software v10.10.1.1 and saved in.fep format. The Feature Extraction software also checks the quality of aCGH by measuring Derivative Log_2_ Ratio Standard Deviation (DLRSD), Signal-To-Noise Ratio (SNR) and Background Noise (BGNoise). The data were analyzed with Agilent Genomic Workbench 5.0 software. In each array spot log_2_ ratios of Cy3 versus Cy5 were computed with the default *P*-value threshold 0.05 and overlap threshold value 0.9. The CNVs were represented by gains and losses of normalized fluorescence intensities relative to the reference and called by conservative criteria which required alterations of >0.5 log_2_ ratios over 5 neighboring probes. Homozygous losses were called when signal log_2_ ratio was <−2.0. Copy number variable regions (CNVRs) were determined by ADM-2 algorithm [91] by combining overlapping and adjacent CNVs in all samples across the CGH experiments. Output files were generated with genomic coordinates and cytoband locations for all CNVs. The raw data were submitted to NCBI Gene Expression Omnibus (GEO) accession GSE55266.

### Array performance evaluation

To evaluate baseline variations and determine FDR [92], [93] female and male self-to-self, and female-to-male control hybridizations were conducted using blood DNA from one female and one male Thoroughbred horses. The female Thoroughbred, *Twilight*, was the DNA donor for the horse reference sequence EquCab2 [56] and the origin of the probes on the tiling array. The male Thoroughbred, *Bravo*, a half-sibling to *Twilight*, was the DNA donor for the CHORI-241 BAC library (http://bacpac.chori.org/equine241.htm) and the origin of all Y chromosome probes on the array. The FDR was calculated as a percentage of the ratio of CNVs in self-to-self hybridization to the total number of CNVs in all experiments. Additionally, array performance was evaluated by self-to-self hybridizations with blood and hair DNA from one Quarter Horse (H528, Table S1). Hybridization quality was assessed by DLRSD which calculates probe-to probe log ratio noise of an array; (http://www.chem.agilent.com/Library/applications/5989-6624EN.pdf): DLRSD <0.2 was considered excellent; 0.2≥DLRSD≤0.3 was good, and values >0.3 indicated poor quality hybridization.

### Chromosome CNVR enrichment

Horse chromosome enrichment percentage was determined by the total length of CNVRs present in each chromosome, divided by chromosome length (Ensembl, http://www.ensembl.org/index.html).

### Gene ontology enrichment analysis

Ensembl gene list (Ensembl Genebuild 73.2) along with their position in the horse genome was added to Agilent Genomic Workbench as a custom track to determine the genic and intergenic CNVs. Gene Ontology analysis (GO) and Kyoto Encyclopedia of Genes and Genomes (KEGG) pathway analysis of the genes present in CNVs were performed using DAVID bioinformatics tool with default settings [94], [95]. Because only a limited number of genes in the horse genome have been annotated, horse gene IDs were converted to orthologous human Ensembl gene IDs by BioMart, followed by GO and pathway analyses, as described above. Biological functions of the genes in CNVRs were further analyzed manually by data mining in Ensembl (http://www.ensembl.org/index.html), UCSC (http://genome.ucsc.edu/) and NCBI (http://www.ncbi.nlm.nih.gov/) Genome Browsers searching for data for equine orthologs in other mammalian species. CNVs present in intergenic regions were analyzed in UCSC genome browser and NCBI and GeneCards (http://www.genecards.org/) for similarities to known mammalian genes.

A composite CNV dataset for the horse (Table S10) was generated by aligning genomic positions of CNVs/CNVRs from this and all previously published studies [35], [36], [37], [38], [39]. Partially or completely overlapping and adjacent CNVs (the end position of a previous CNV and the start position of the next CNV are the same) were consolidated into one CNVR.

### Array CGH data validation by qualitative and quantitative PCR

Genomic copy number changes as detected by aCGH were validated by quantitative PCR (qPCR) for 18 selected CNVRs using 22 probe-specific primers. Additionally, 8 putative homozygous deletions were validated by regular (qualitative) PCR. Primers (Table S2) were designed inside CNVRs using array probe sequences and the horse whole genome sequence information (EquCab2 at UCSC: http://genome.ucsc.edu/and Ensembl: http://www.ensembl.org/index.html) and Primer3 software (http://bioinfo.ut.ee/primer3-0.4.0/primer3/input.htm). The qPCR experiments were performed with LightCycler 480 (Roche Diagnostics) in triplicate assays. Each assay was done in triplicate 20 µL reactions containing 50 ng of template DNA, 10 µM primers and the SYBR Green PCR kit (Roche). Relative copy numbers of the selected regions were determined in comparison to the reference sample (Thoroughbred and Quarter Horse) and normalized to an autosomal reference gene *GAPDH*. The cycling conditions were 1 cycle 5 min at 95°C; 45 cycles 10 sec at 95°C, 5 sec at 58°C, and 10 sec at 72°C; 1 cycle for melting curve 30 sec 95°C, 30 sec 65°C and final cooling 20 sec at 50°C. Quantification of the copy number was carried out using the comparative C_T_ method (2^ΔΔ^Ct) [96], [97] with p<0.05 as a cut-off threshold for statistical significance. Qualitative PCR results were analyzed by agarose gel electrophoresis.

### Array CGH data validation by fluorescence in situ hybridization (FISH)

CNV specific primers were used to screen CHORI-241 BAC library (http://bacpac.chori.org/equine241.htm) by PCR (Table S2); BAC DNA was isolated by Plasmid Midiprep kit (Qiagen), labeled with biotin-16-dUTP or digoxigenin-11-dUTP using Biotin- or DIG-Nick Translation Mix (Roche), and hybridized to metaphase chromosomes of CNV carriers and control horses following standard protocols [98]. A BAC clone representing a non-CNV region was used as a control in each FISH experiment. Images for a minimum of 20 metaphase and/or interphase cells were captured for each experiment and analyzed with a Zeiss Axioplan2 fluorescent microscope equipped with Isis v5.2 (MetaSystems GmbH) software.

### Phylogenetic analysis

Genotypes for 15 microsatellite loci [74]; E.G. Cothran, unpublished) were available for 32 out of 41 horse breeds involved in CNV studies (see Table S12). Majority-rule consensus of Restricted Maximum Likelihood (RML) trees were constructed and visualized as described elsewhere [74]. The Przewalski Horse population was used as an out-group.

## Supporting Information

Figure S1 Array and aCGH quality control. **A**. Genome-wide distribution of CNVs in self-to-self hybridization (upper) compared to cumulative hybridizations with all animals (lower) to determine FDR; green vertical lines denote CNVs; **B**. Male-to-female aCGH results for the X chromosome; **C**. DLRSD values of aCGH using DNA from blood (left) and from hair (right) of the same individual.
(PDF)

Figure S2 Homozygous deletions. Confirmation of putative homozygous deletion CNVs (red arrows) by qualitative PCR.
(PDF)

Figure S3 Validation of selected CNVRs by quantitative PCR (qPCR). **A1–A14** qPCR was in agreement with aCGH in discovery horses and their breed-mates; **B1–B3** qPCR agrees with aCGH in the discovery horse (left) but not in additional horses of the same breed (right).
(PDF)

Figure S4 Validation of a copy number gain in chr1 (114.0 Mb) by FISH. **A**. and **B**. – metaphase and interphase of the Thoroughbred control; **C**. and **D**. metaphase and interphase of a Quarter Horse; red signals - BAC 132B13; green signals in **D**. – a single-copy control BAC. Note the difference in copy numbers between homologous chromosomes in both horses.
(PDF)

Table S1 Horse breeds (n = 16) and individuals (n = 38) used in this study.
(XLSX)

Table S2 Primers for quantitative and qualitative PCR to validate CNVs.
(XLSX)

Table S3 List of all 950 CNV calls in the study cohort.
(XLSX)

Table S4 Tentative breed-specific CNVRs.
(XLSX)

Table S5 258 CNVRs identified in the horse genome in this study.
(XLSX)

Table S6 Gains and losses with high log_2_ alteration values.
(XLSX)

Table S7 Genomic locations, names, symbols and known or predicted functions of copy number variable genes.
(XLSX)

Table S8 Intergenic CNVRs.
(XLSX)

Table S9 GO analysis of equine copy number variable genes.
(XLSX)

Table S10 Integrated dataset of 1476 CNVs/CNVRs in the horse.
(XLSX)

Table S11 Details of validation of 19 selected CNVRs by qPCR.
(XLSX)

Table S12 List of horse breeds studied for CNVs.
(XLSX)
