# Supplementary material for: Copy Number Variation in the Horse Genome
Source: PLoS Genet. 2014 Oct 23;10(10):e1004712. doi: 10.1371/journal.pgen.1004712 (PMC4207638; doi:10.1371/journal.pgen.1004712)
Supplement: Figure S1 — Array and aCGH quality control. A. Genome-wide distribution of CNVs in self-to-self hybridization (upper) compared to cumulative hybridizations with all animals (lower) to determine FDR; green vertical lines denote CNVs; B. Male-to-female aCGH results for the X chromosome; C. DLRSD values of aCGH using DNA from blood (left) and from hair (right) of the same individual. (PDF) [file pgen.1004712.s001.pdf]

A. Self-self hybridization

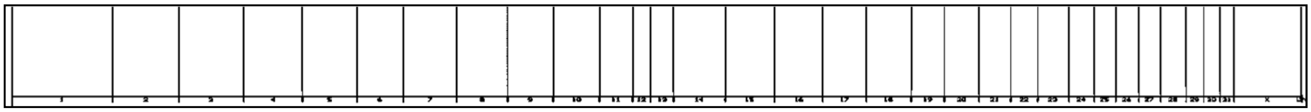

Thoroughbred 2 self-self hybridization

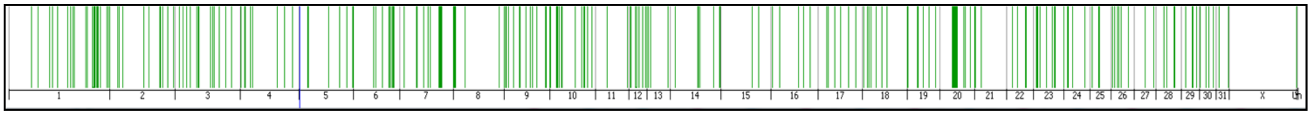

Cumulative hybridizations with all 36 animals

B. Male-to-female aCGH for the X chromosome

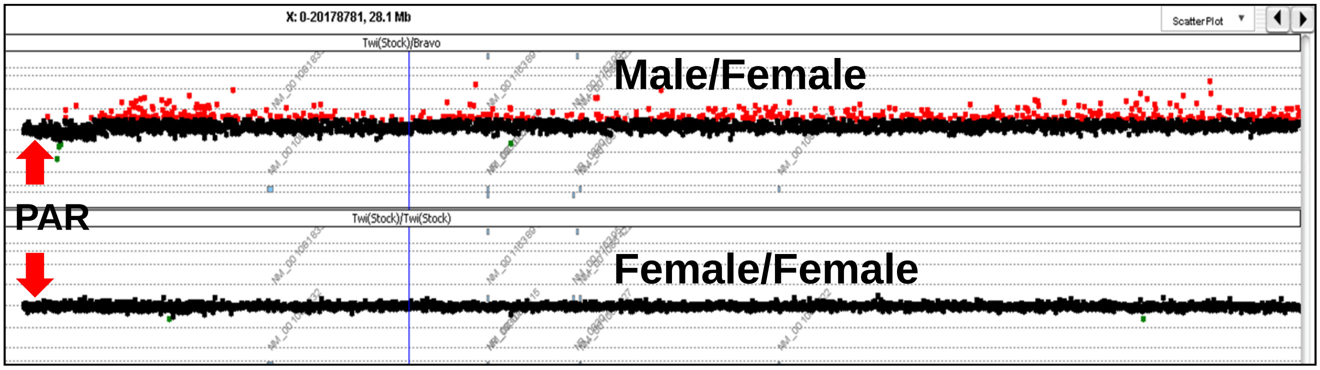

C. DNA quality check

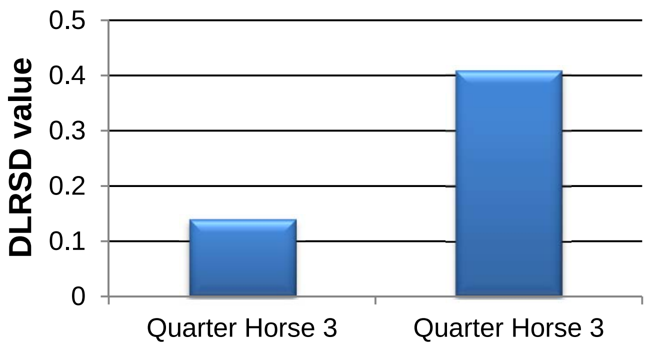

Figure S1. Array and aCGH quality control: A. Genome-wide distribution of CNVs in self-to-self hybridization (upper) compared to cumulative hybridizations with all animals (lower) to determine FDR; green vertical lines denote CNVs; **B**. Male-to-female aCGH results for the X chromosome; **C.** DLRSD values of aCGH using DNA from blood (left) and from hair (right) of the same individual
