# Supplementary figures and images for: Copy Number Variation in the Horse Genome
Source: PLoS Genet. 2014 Oct 23;10(10):e1004712. doi: 10.1371/journal.pgen.1004712 (PMC4207638; doi:10.1371/journal.pgen.1004712)

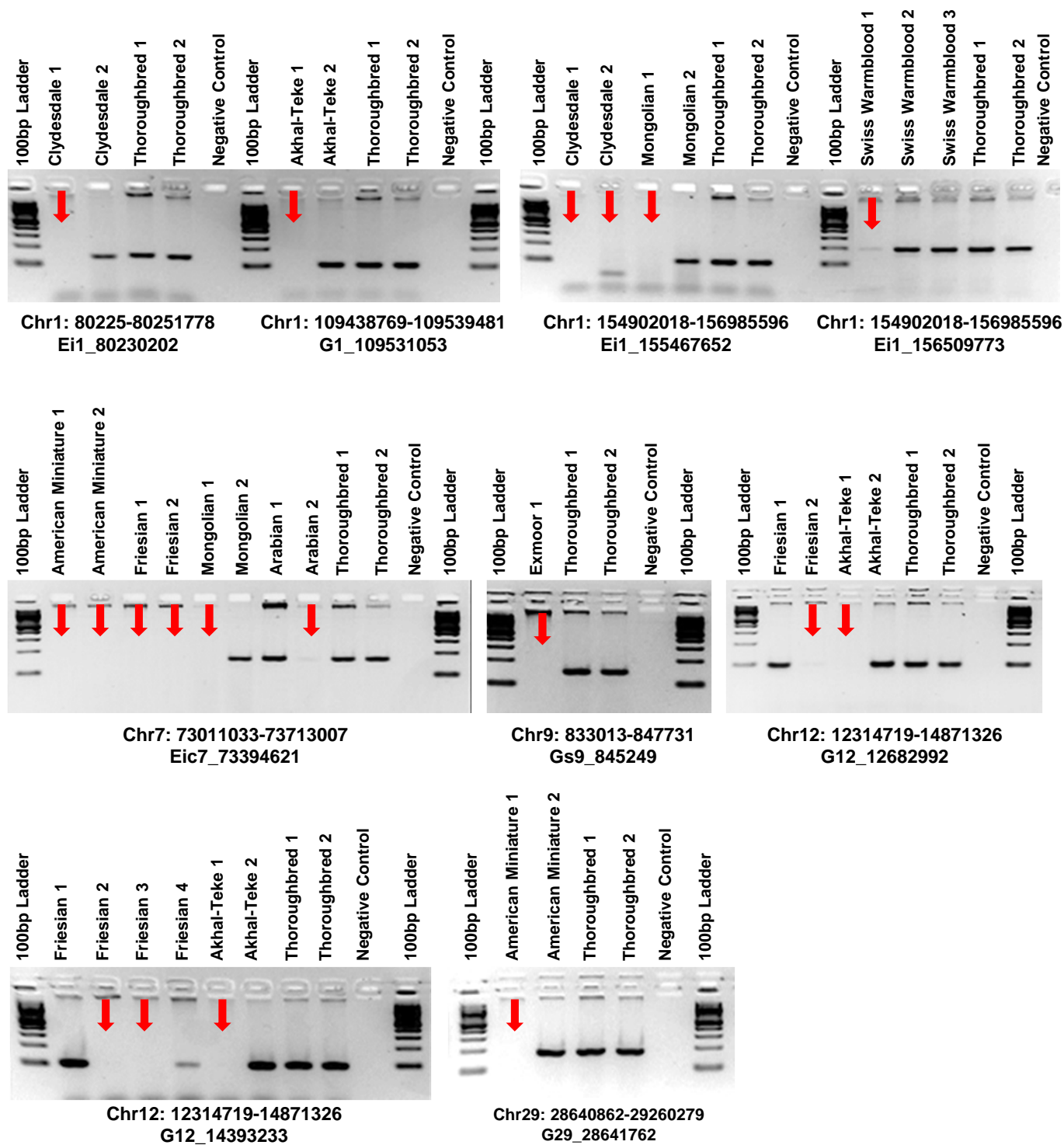

**Figure S2:** Confirmation of putative homozygous deletion CNVs (red arrows) by qualitative PCR

Supplement: Figure S2 — Homozygous deletions. Confirmation of putative homozygous deletion CNVs (red arrows) by qualitative PCR. (PDF) [file pgen.1004712.s002.pdf]
