# Supplementary material for: Copy Number Variation in the Horse Genome
Source: PLoS Genet. 2014 Oct 23;10(10):e1004712. doi: 10.1371/journal.pgen.1004712 (PMC4207638; doi:10.1371/journal.pgen.1004712)
Supplement: Figure S3 — Validation of selected CNVRs by quantitative PCR (qPCR). A1–A14 qPCR was in agreement with aCGH in discovery horses and their breed-mates; B1–B3 qPCR agrees with aCGH in the discovery horse (left) but not in additional horses of the same breed (right). (PDF) [file pgen.1004712.s003.pdf]

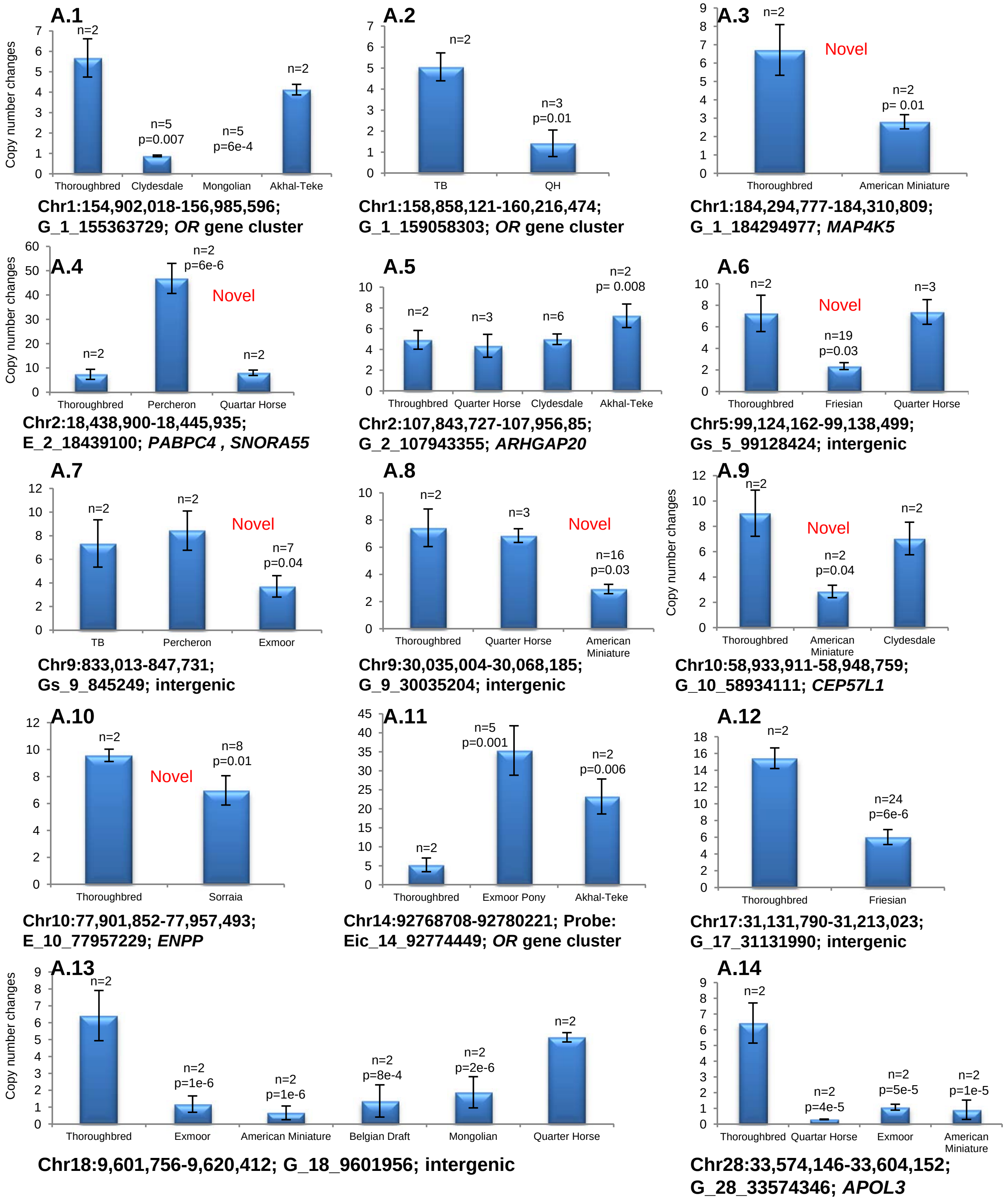

Figure S3. Validation of selected CNVRs by quantitative PCR (qPCR): A1-A14
qPCR was in agreement with aCGH in discovery horses and their breed-mates.

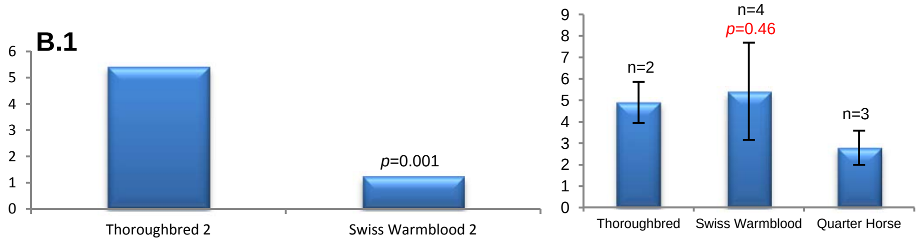

Chr7:74,885,505-74,911,413; G_7_74887990; OR gene cluster

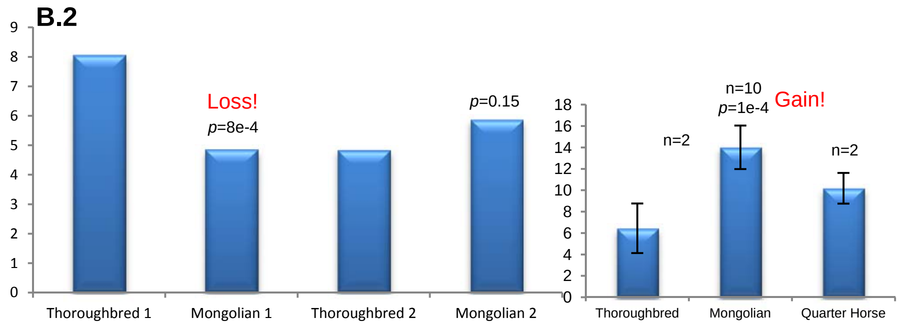

Chr17:18,834,382-19,076,884; G_17_18840001; *TPTE2, THSD1, VPS36*

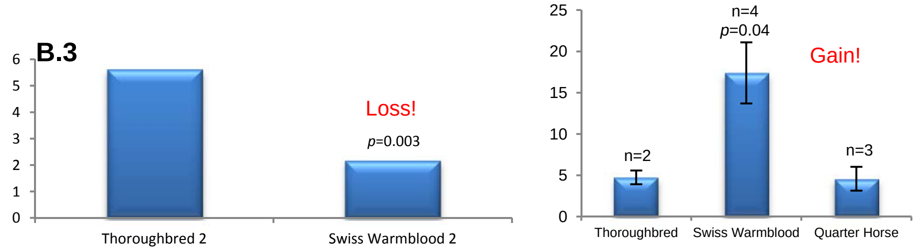

Chr20:32,093,989-32,437,216; G_20_32208360; pseudogene

Figure S3. Validation of selected CNVRs by quantitative PCR (qPCR): B1-B3
qPCR agrees with aCGH in the discovery horse (left) but not in additional horses of the same breed (right).
