# Supplementary material for: Copy Number Variation in the Horse Genome
Source: PLoS Genet. 2014 Oct 23;10(10):e1004712. doi: 10.1371/journal.pgen.1004712 (PMC4207638; doi:10.1371/journal.pgen.1004712)
Supplement: Figure S4 — Validation of a copy number gain in chr1 (114.0 Mb) by FISH. A. and B. – metaphase and interphase of the Thoroughbred control; C. and D. metaphase and interphase of a Quarter Horse; red signals - BAC 132B13; green signals in D. – a single-copy control BAC. Note the difference in copy numbers between homologous chromosomes in both horses. (PDF) [file pgen.1004712.s004.pdf]

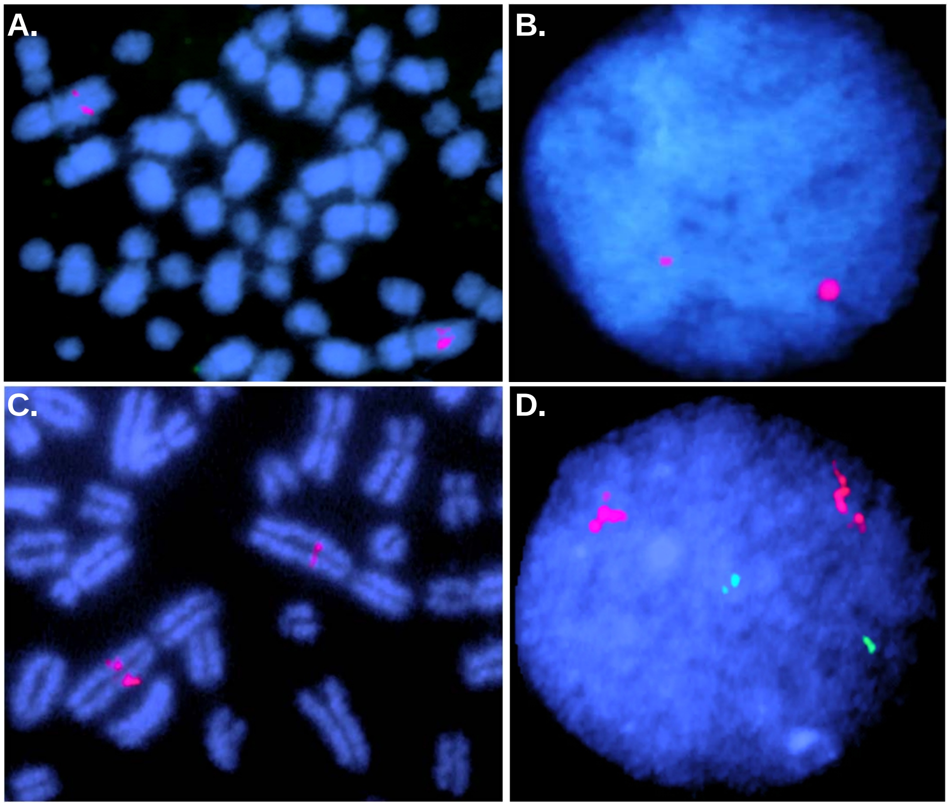


**Figure S4. Validation of a copy number gain in chr1 (114.0 Mb) by FISH: A.** and **B. –** metaphase and interphase of the Thoroughbred control; **C.** and **D.** metaphase and interphase of a Quarter Horse; red signals - BAC 132B13; green signals in **D.** – a single-copy control BAC. Note the difference in copy numbers between homologous chromosomes in both horses.
